# Dissemination and outcome reporting bias in clinical malaria intervention trials: a cross-sectional analysis

**DOI:** 10.1186/s12936-024-05115-6

**Published:** 2024-09-30

**Authors:** Lydia Pool, Claire Ruiz del Portal Luyten, Rob W. van der Pluijm, Patrick Soentjens, Thomas Hanscheid, Martin P. Grobusch, Benjamin J. Visser

**Affiliations:** 1 Center of Tropical Medicine and Travel Medicine, Department of Infectious Diseases, Amsterdam University Medical Centers, University of Amsterdam, Amsterdam, the Netherlands; 2 Université Paris Cité, G5 Infectious Disease Epidemiology and Analytics, Institut Pasteur, 75015 Paris, France; 3 Department of Clinical Sciences, Institute of Tropical Medicine (ITM), Antwerp, Belgium; 4 Faculdade de Medicina, Universidade de Lisboa, Lisbon, Portugal; 5 Masanga Medical Research Unit (MMRU), Masanga, Sierra Leone; 6 Centre de Recherches Médicales en Lambaréné (CERMEL), Lambaréné, Gabon; 7 Institute of Tropical Medicine & Deutsches Zentrum Für Infektionsforschung, University of Tübingen, Tübingen, Germany; 8 Institute of Infectious Disease and Molecular Medicine, University of Cape Town, Cape Town, South Africa

**Keywords:** Malaria research trials, ClinicalTrials.gov, Non-dissemination, Publication bias, Dissemination bias, Publication bias, Outcome reporting bias, Trial registration, WHO dissemination standards

## Abstract

**Background:**

Dissemination and outcome reporting biases are a significant problem in clinical research, with far-reaching implications for both scientific understanding and clinical decision-making. This study investigates the prevalence of dissemination- and outcome reporting biases in registered interventional malaria research.

**Methods:**

All malaria interventional trials registered on ClinicalTrials.gov from 2010 to 2020 were identified. Subsequently, publications that matched the registration were searched. The primary outcome measures were the percentage of registered studies that resulted in subsequent publication of study results, the concordance between registered outcomes, and reported outcomes. Secondary outcomes were compliance with WHO standards for timely publication (issued in 2017) of summary study results in the respective trial registry (within 12 months of study completion) or peer-reviewed publication (within 24 months of study completion) was evaluated.

**Results:**

A total of 579 trials were identified on ClinicalTrials.gov, of which 544 met the inclusion criteria. Notably, almost 36.6% of these trials (199/544) were registered retrospectively, with 129 (23.7%) registered after the first patient enrolment and 70 (12.9%) following study completion. Publications were identified for 351 out of 544 registered trials (64.5%), involving 1,526,081 study participants. Conversely, publications were not found for 193 of the 544 registrations (35.5%), which aimed to enrol 417,922 study participants. Among these 544 registrations, 444 (81.6%) did not meet the WHO standard to post summary results within 12 months of primary study completion (the last visit of the last subject for collection of data on the primary outcome), while 386 out of 544 registrations (71.0%) failed to publish their results in a peer-reviewed journal within 24 months of primary study completion. Discrepancies were noted in the reported primary outcomes compared to the registered primary outcomes in 47.6% (222/466) of the published trials, and an even higher discordance rate of 73.2% (341/466 publications) for secondary outcomes.

**Conclusions:**

Non-dissemination remains a significant issue in interventional malaria research, with most trials failing to meet WHO standards for timely dissemination of summary results and peer-reviewed journal publications. Additionally, outcome reporting bias is highly prevalent across malaria publications. To address these challenges, it is crucial to implement strategies that enhance the timely reporting of research findings and reduce both non-dissemination and outcome reporting bias.

**Supplementary Information:**

The online version contains supplementary material available at 10.1186/s12936-024-05115-6.

## Background

Non-publication and delayed publication of clinical trial results, collectively termed 'dissemination bias', along with outcome reporting bias poses significant challenges to evidence-based medicine, including infectious diseases (see Box 1 for definitions) [1, 2]. Dissemination bias contributes to a skewed perception of evidence and can lead to overestimated treatment effectiveness and misrepresented side effects, thereby adversely affecting both scientific research and clinical practice [3–5]. For example, an estimated 50% of outcomes from randomized controlled trials remain unpublished, notably even introducing bias into systematic reviews [6, 7]. Reporting is sometimes entirely lacking [4] or incomplete, or inconsistent; frequently diverging from initial protocols [7, 8]; or showing poor adherence to predefined trial outcomes [9, 10]. Factors such as funding sources, the pressure to publish positive results, vested interests in specific treatments, and the geographical locations of trials contribute to this issue [11, 12]. There is a growing recognition of the importance of transparently sharing research results both timely and comprehensively [7, 8, 13, 14]. To enhance transparency, accountability, and reproducibility, it is crucial to publish all study protocols and results (positive or negative); thus preventing duplication of trials, misuse of data, and wasting of research funds, whether public or private [7, 15]. Researchers, authors, sponsors, editors, and publishers all have an ethical obligation towards trial participants concerning timely dissemination of complete and accurate research results [16–18]. Adhering to best practices in study registration and reporting is essential for advancing evidence-based medicine [19, 20]. According to the Declaration of Helsinki (2013), every research study involving human subjects must be registered in a publicly accessible database before the recruitment of the first participant. The declaration also mandates the timely dissemination of results and stipulates that negative or inconclusive findings should be publicly available [21].

**Box 1:** Definitions of terms used

Dissemination of results: The process of making research findings available to the public through any medium, including online platforms and databases, also described as ‘reporting event’ in this review
Peer-reviewed publication of results: The publication of study results in a scholarly journal that employs a formal peer-review process to evaluate submissions
WHO timely dissemination (12-Month Timeframe): The requirement that summary results of studies be available in registries within 12 months following study completion (without peer review)
WHO timely dissemination (24-Month Timeframe): The requirement that study results be published in a peer-reviewed journal within 24 months following study completion
Dissemination bias: Selective dissemination (delayed or non-dissemination) of results depending on the type and direction of the results, can apply to any form of result dissemination, not only journal publications
Outcome reporting bias: The selective reporting of study outcomes, including changing outcome definitions, prioritizing certain results over others, or altering the order of primary and secondary outcomes based on the findings

Research increasingly focuses on identifying various forms of biases and examining the effects of trial registration across different fields [7]. However, to date, no study has specifically addressed the prevalence of non-timely reporting and outcome reporting bias in malaria research, leaving the impact on patient care and the broader research landscape unquantified. This gap is particularly concerning, given the limited funding for malaria research and the substantial global burden of the disease, with 249 million cases and 608,000 deaths reported in 2022 [22]. It is known that pharmaceutical industry sponsorship impacts research outcomes and quality, often introducing systematic bias that favours the sponsor's products [23]. Additionally, some argue that evidence-based medicine is compromised due to biased trials and selective publication driven by industry funding [24]. Malaria research may also exhibit patterns of bias; however, it could be argued that it is less influenced by large commercial and pharmaceutical interests, potentially resulting in reduced bias. This study aims to investigate the magnitude of biases within the field of malaria research. Specifically, the primary objectives are to estimate the proportion of registered interventional malaria trials that remain unpublished and to assess the prevalence of outcome reporting bias by examining the discordance between registered and published outcomes. The secondary objective is to estimate the proportion of registered interventional malaria trials that are disseminated within the WHO-recommended timelines.

## Methods

This observational cross-sectional study focused exclusively on interventional malaria trials, conducted in malaria endemic and non-endemic regions, reflecting their significant impact on patient care [7, 8, 10, 19]. STROBE reporting guidelines for cross-sectional studies were followed [25] (see STROBE checklist in Supplementary file 1). This study is an observational cross-sectional study of malaria trials registered on www.ClinicalTrials.gov, a major database of clinical studies conducted around the world [26]. This study did not receive internal or external funding [27]. Selection criteria included trials that were first posted from January 1, 2010, to January 1, 2020, specifying 'malaria' (including *Plasmodium falciparum, Plasmodium vivax, Plasmodium malariae, Plasmodium ovale,* and *Plasmodium knowlesi*) as the condition/disease and restricted to interventional clinical trials. Only interventional malaria trials were eligible. Interventional trials, as defined by ClinicalTrials.gov, are a type of clinical study in which participants are assigned to groups receiving one or more interventions (or no intervention) to evaluate the effects on biomedical or health-related outcomes. The group assignments are determined by the study protocol, and participants may receive diagnostic, therapeutic, or other forms of intervention. Data were downloaded on November 15, 2022. A completion date of July 1, 2021, was set to allow enough time for the publication of the registered trials. Publications corresponding to trials registered on ClinicalTrials.gov were identified using searches in PubMed, Google Scholar, EMBASE, and other search engines; utilizing registration numbers, titles, and researchers’ names. The searches also included checks for results posted on ClinicalTrials.gov, and were last updated on January 24, 2024, using tools such as ChatGPT4 and Perplexity.ai for assistance. Online pre-print servers such as medRxiv were not included in the search. A ‘reverse search’ in PubMed was performed to identify all PubMed-indexed malaria clinical trials using the Medical Subject Headings (MeSH) term ‘malaria’ with a ‘clinical trial’ filter, covering the period from January 1, 2018, to January 1, 2024. Full texts of search results were reviewed to assess their association with an NCT registration.

If no publication was found, the first or corresponding author of the trial was contacted via email or ResearchGate to inquire about reasons for non-publication or delays. If no response was received after the initial contact, two additional follow-up emails were sent at intervals of 7–10 days. Trials that received no response were classified as unpublished. Additional details, including the identification of subsequent publications and analysis of publication bias, are provided in Supplementary File 1. The analysis of dissemination bias focused on whether registered research was eventually published and, if so, the duration between the primary completion date and the publication of summary results on the trial registry or publication of a peer-reviewed journal publication.

Registrations were downloaded from ClinicalTrials.gov, selecting interventional studies across all age groups, sexes, recruitment statuses, and results (including those with and without posted study results). The number of enrolled participants and the time from study completion to publication were calculated. The 'Timing of registration' was determined by comparing the 'first posted' date with the ‘start trial’ date, categorized as follows: (1) ‘registered before’ the trial started (category I); (2) ‘same date’ as the trial start (category II); (3) ‘in between’ the start and completion dates (category III); and (4) ‘after completion’ of the trial (category IV). The median time from trial completion to publication was initially calculated for the first publication per registered trial (online first date). Subsequently, trials published before registration or completion, and those registered after the trial had started (categories III and IV), were excluded. This adjustment accounts for the still common practice of registering studies post-completion to fulfil formal requirements (‘retrospective registration’). For four registrations, there was no completion date; in those four cases, the ClinicalTrials.gov registered ‘primary completion date’ was used as alternative. Further details on variables and analyses are available in Supplementary file 1.

**Fig. 1:**
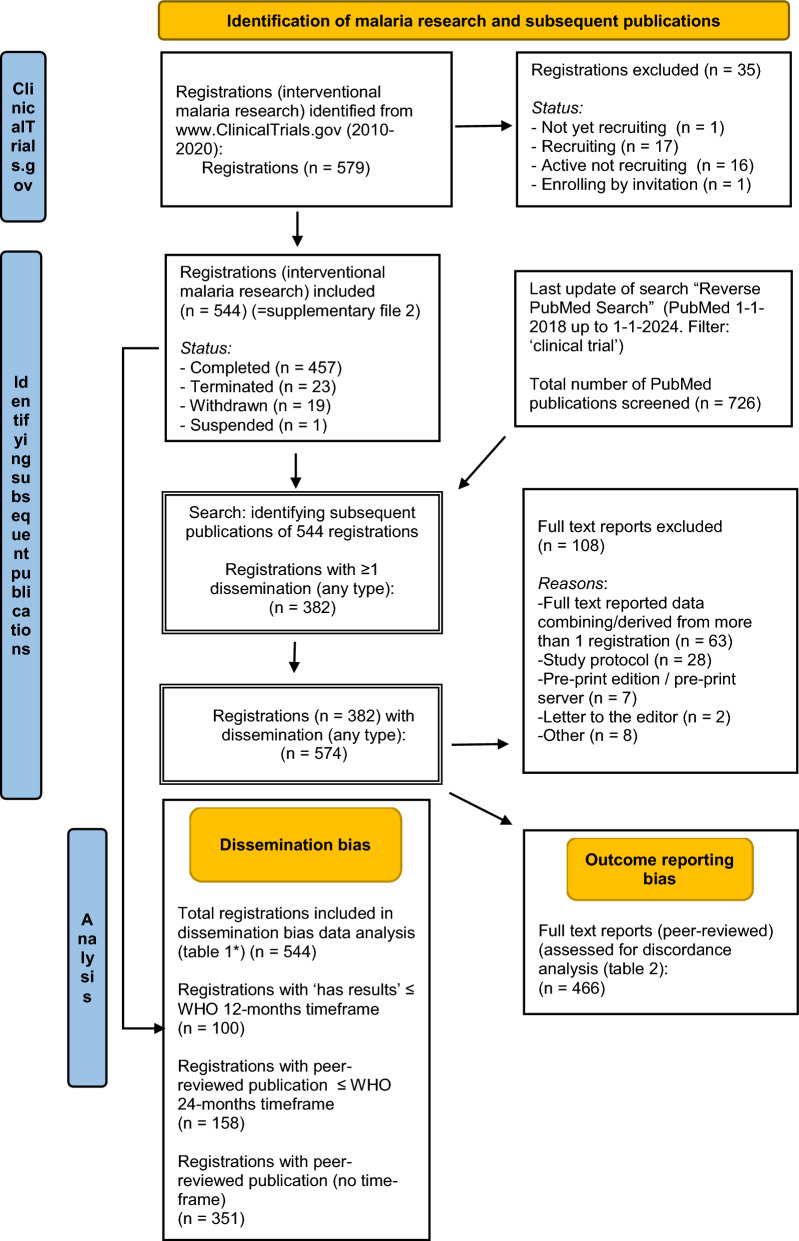
Flow chart of the analysis of registered trials with dissemination and outcome reporting bias. The number of registrations with peer-reviewed publications (n = 351) as shown in Table 1, column (D) differs from the number of registrations with any type of dissemination (n = 382) (see double-lined boxes) because it includes non-peer-reviewed results, posters, published study protocols etc.)

The analysis focused on identifying registrations that exhibited either dissemination bias or outcome reporting bias, as detailed in the flow chart (Fig. 1). If a single NCT number corresponded to one or more publications, all relevant publications were included in the outcome reporting bias analysis. However, publications referencing multiple NCT numbers were excluded from the analysis of outcome reporting bias. This exclusion is due to the difficulty in accurately determining discrepancies between reported outcomes and those registered, as such publications compiling data from multiple trials.

Based on the World Health Organization (WHO) guidelines issued mid-2017, which endorse the timely publication of research results, within 12 months in registries for non-peer-reviewed results, and within 24 months in peer-reviewed journals following the primary study’s completion [28] the prevalence of non-publication and the adherence to these publication standards in the registered interventional malaria clinical trials was assessed. The main goal of this assessment was to evaluate the feasibility of adhering to WHO publication timelines when applied to interventional malaria research and to determine whether these recommendations had any impact on dissemination bias following their release.

To evaluate outcome reporting bias, this study compared the primary, secondary, and other outcomes listed in the trial registrations with those reported in corresponding publications to detect any discrepancies. Publications which did not feature original data pertaining to the specific NCT registration number, entirely lacked results, or included findings from multiple registered trials, (making it difficult to conduct comparisons between registered and published outcomes) were excluded (see flow chart, Fig. 1). The levels of concordance were categorized as 'complete concordance', 'complete discordance' or ‘partly concordant’ (for more details on definitions, see Supplementary file 1). This analysis intentionally did not assess the validity of the reasons for outcome changes to avoid subjectivity, focusing instead on the presence of discrepancies. While discrepancies between registered and published outcomes were noted, the study did not list publications that deviated from their registrations to prevent potential damage to researchers' reputations and avoid judging deviations from registered protocols. This approach recognizes that there may be valid, undisclosed reasons for these discrepancies, and prioritizes the broader goal of enhancing reporting quality and reducing outcome reporting bias over individual accountability.

## Results

From a total of 579 NCT interventional malaria trial registrations retrieved, 544 met the inclusion criteria (Fig. 1 and Table 1, Supplementary file 2). Most registered trials, (441/544, 81%), focused on (anti-malarial) drugs or biologicals (including vaccines), with approximately half, (282/544, 51.8%), solely conducted in Africa. Additionally, 120 trials (22%) took place in South America, Asia, or Southern Africa, and 142 trials (26.1%) occurred in the USA, Europe, or Australia. Only 22.7% (124/544) of the studies referred to some form of industry as funder, and 15.6% (85/544) listed ‘pharmacy’ (pharmaceutical industry) as the sponsor (see Table S1, Supplementary file 1). Hundred and twenty-four trials (22.8%) included only children as study participants. The majority of trials (244, 44.9%) had an anticipated enrolment of less than 100 participants, while 26 trials (4.8%) intended to enrol more than 10,000 participants. Further information and details of these trials can be found in the supplementary file 1 (Table S1). Retrospective registration is quite common in malaria research, with 199 out of the 544 trials (37%) registered retrospectively, as indicated by categories III and IV (Table 1). This trend has remained relatively constant over the years (Fig. S2—Supplementary File 1). However, there was a slight relative increase in recent years (2017–2019), although the total number of registrations in these years was also much less than those in previous years. A linear regression model indicated that there was no statistically significant difference (*p* = *1,0* for time-coefficient) in the registration rates and non-timely dissemination of summary trial results (12 months’ time-frame) or peer-reviewed publications (24 months’ timeframe) before and after June 2017.

**Table 1:** Registered malaria trials and dissemination/publications

	(A) Total	(B) Timely dissemination (WHO: 12 months)	(C) Timely publication (WHO: 24 month)	(D) Total published (no timeframe)
Registrations on ClinicalTrials.gov (n)	544	100 18.4% of 544	158 29.0% of 544	351 64.5% of 544
Trial status				
Completed	457 (84.0%)	85 (85.0%)	142 (89.9%)	316 (90.0%)
Terminated	23 (4.2%)	8 (8.0%)	5 (3.2%)	15 (4.3%)
Withdrawn	19 (3.5%)	1 (1.0%)	1 (0.6%)	1 (0.3%)
Suspended	1 (0.2%)	1 (1.0%)	1 (0.6%)	1 (0.3%)
Unknown	44 (8.1%)	5 (5.0%)	9 (5.7%)	18 (5.1%)
Timing of registration				
Before start of trial (category I)	248* (45.6%)	53 (53.0%)	75 (47.5%)	165 (47.0%)
Date same as start of trial (category II)	97* (17.8%)	17 (17.0%)	23 (14.6%)	58 (16.5%)
After start of trial (category III)	129 (23.7%)	24 (24.0%)	44 (27.8%)	90 (25.6%)
After completion of trial (category IV)	70 (12.9%)	6 (6.0%)	16 (10.1%)	38 (10.8%)
Geography (location of the trial)				
Africa	282 (51.8%)	59 (59.0%)	97 (61.4%)	197 (56.2%)
Europe/US/Australia	142 (26.1%)	18 (18.0%)	27 (17.1%)	78 (22.2%)
Rest of the world	120 (22.1%)	23 (23.0%)	34 (21.5%)	76 (21.6%)
Secondary outcomes				
Yes	486 (89.3%)	92 (92.0%)	149 (94.3%)	329 (93.7%)
No	58 (10.7%)	8 (8.0%)	9 (5.7%)	22 (6.3%)
Funder				
Industry	124 (22.8%)	27 (27.0%)	25 (15.9%)	78 (22.3%)
Non-industry	420 (77.2%)	73 (73.0%)	133 (84.1%)	273 (77.7%)
Sponsor				
Industry	85 (15.6%)	21 (21.0%)	19 (12.1%)	57 (16.3%)
Non-industry	459 (84.4%)	79 (79.0%)	139 (87.9%)	294 (83.7%)
Intervention type				
Drug/biological	441 (81.1%)	86 (86.0%)	127 (80.4%)	283 (80.7%)
Non-drug/biological	103 (18.9%)	14 (14.0%)	31 (19.6%)	68 (19.3%)
Study duration				
< 1 year	175 (32.2%)	12 (12.0%)	30 (19.0%)	90 (25.6%)
< 2 year	188 (34.6%)	30 (30.0%)	52 (32.9%)	128 (36.5%)
< 3 year	90 (16.5%)	19 (19.0%)	33 (20.9%)	66 (18.8%)
> 3 year	87 (16.0%)	39 (39.0%)	43 (27.2%)	65 (18.5%)

Information from all included trials registered on ClinicalTrials.gov (n = 544) in column (A). Timely dissemination of any type of result with 12 months (B) or peer-reviewed publication within 24 months (C) as per WHO standard. Column (D) any peer reviewed publication without timeline

Percentages refer to (n) in first row: registrations on clincialtrials.gov. Sum of the numbers marked with * (248 + 97 = 351, column A, timing of registration I and II) used in Table 2. Funder ‘Industry’ compiled from 3 categories: ‘industry + industry/other + US/industry’. Sponsor ‘Industry’ compiled from 1 category: ‘pharmacy’. Interventions type compiled from categories ‘drug’ + ‘biological’ versus non-drugs/biological: ‘dietary + ’other’ + ‘procedure/behaviour/device’

Study duration: For 4 trials out of 544 (0.7%) there was a missing completion date. Further information on registered trials can be found in detailed table S1 in Supplementary file 1

No peer-reviewed journal publications were found for over one-third of the trial registrations (35.5%; 193/544). Of these 193 trial registrations without peer-reviewed publications, attempts were made to contact the authors via email or social media for 95 of them, after eliminating 98 cases for which current contact information was unavailable. Out of these, 30 researchers responded (response rate: 31.6%), often mentioning reasons for non-publication such as recruitment delays, funding issues, non-efficacy of the intervention, serious adverse events, or complications related to COVID-19. Dissemination bias analysis showed that most registered trials (81.6%; 444/544) failed to meet the WHO standard of disseminating results within 12 months of study completion (see Fig. 1 and Table 1). Further analysis indicated that 65% of registered trials (351/544) had at least one peer-reviewed publication at some point. However, only 29% of trials (158/544) achieved this within the 24-month timeframe specified by the WHO standard. The issuance of the WHO joint statement mid-2017 appeared to have no observable effect on publication timelines (see Table 2). A sensitivity analysis (chi-square test, see Supplementary File 1) showed no significant differences in dissemination versus non-dissemination (without time limits) between Categories I and II compared to Categories III and IV. The latter two categories were excluded from the WHO timely dissemination analysis.

**Table 2:** Publication Timeliness Before and After 2017 WHO Endorsement

	Trial registrations total (n = 345)	Trial registrations before June 2017 (n = 288)	Trial registrations after June 2017 (n = 57)
Registered before start of the trial (Cat. I)	248 (71.9%)	204 (70.8%)	44 (77.2%)
Registered on day of the trial start (Cat. II)	97 (28.1%)	84 (29.2%)	13 (22.8%)
Non-timely dissemination (summary results posted on ClinicalTrials.gov)—WHO: 12 months (Cat. I)	275 (79.7%)	233 (80.9%)	42 (73.7%)
Non-timely dissemination (summary results posted on ClinicalTrials.gov)—WHO: 12 months (Cat. II)	70 (20.3%)	55 (19.1%)	15 (26.3%)
Non-timely publication—(peer-reviewed journal publication) WHO: 24 months (Cat. I)	247 (71.0%)	206 (71.5%)	41 (71.9%)
Non-timely publication—(peer-reviewed journal publication) WHO: 24 months (Cat. II)	98 (28.4%)	82 (28.5%)	16 (28.1%)

This table includes only those registrations that were either posted on or before the trial start date, categorized as Category I (n = 248) and Category II (n = 97)

Retrospective registrations, falling into Categories III (n = 129) and IV (n = 70), are excluded. The total included is 345 registrations (see Table 1 for details, numbers marked with *)

The dissemination of results within 12 months and peer-reviewed publications within 24 months, are assessed based on their ‘first posted’ date relative to the WHO endorsement mid-2017

The median total duration for all 544 registered trials was 517 days (IQR: 268–853 days). Registered trials with peer-reviewed journal publications (no time frame) had a median duration of 578 days (IQR: 335–974 days), while those without peer-reviewed journal publications (without time-frame) had a median duration of 396 days (IQR: 199–730 days).

The 158 trials that met the WHO’timely dissemination’ definition for peer-reviewed publications within 24 months had a median duration of 685 days (IQR 396–1140 days), while the 386 trial registrations that did not meet this criterium had a median duration of 456 days (IQR 215–738 days). From the completion of the trial (date of the last data collection time point for the last participant visit for the primary outcome measure) to publication in a peer-reviewed journal (no-time frame), the median time was 777 days (IQR: 462–1272 days). The 158 registered trials that met the WHO 24-months’ time-frame had a median study duration of 424 days (IQR 98–592 days). The number of trials that eventually disseminated their results in a peer-reviewed journal publication that published beyond the 24-month WHO time-frame was 193 trials (35% of total registered trials and 54,9% of trial registrations with a peer-reviewed publication without time frame). Registered trials conducted in Africa were more likely to publish their peer-reviewed results within the 24-months’ timeframe compared to trials conducted in the rest of the world (52.4% vs. 30.3% timely publication, respectively; X^2^ = 8.14, n = 544, *p* = *0.004*). There was no difference between the proportion of peer-reviewed results within the 24-months’ timeframe for registered malaria trials funded by the industry (type: ‘funder’, see Table 1) compared to those funded by non-industry sources (25.2% vs. 46.3%, X^2^ = 6.15, n = 544, *p* = *0.013*). When the industry was the ‘sponsor’ of the trial, there was also no difference for the peer-reviewed results were published within the 24-month timeframe, although this difference was not statistically significant (28.7% vs. 43.4%, X^2^ = 2.19, n = 544*, p* = *0.14*). There was no significant difference in intervention type; trials with the intervention type ‘drug’ or biological (e.g., vaccines) did not differ in the proportion of registration to publish their peer-reviewed results within the 24-month timeframe compared to non-drug/biological malaria trials (40.4% vs. 43.0%, X^2^ = 0.06, n = 544, *p* = *0.79*). No difference was observed in meeting the WHO’s 24-month timely publication guidelines for peer-reviewed articles between small to medium-sized malaria trials (< 500 participants) and larger trials (> 500 participants) (X^2^ = 3.11, n = 544 *p* = *0.07*), see Supplementary file 1.

Of the 544 trial registrations, 382 (70%) led to a total of 574 ‘reporting events’ (dissemination in any form). Of these events, 466 (81%) were peer-reviewed journal publications, which were analysed for outcome reporting bias (Fig. 1 and Table 1). As highlighted before, these publications originated from 351 trial registrations, averaging 1.3 publications per registration (ranging from 1 to 14 publications per registration).

In the analysis of outcome reporting bias (Table 3), nearly a quarter of the publications displayed complete discordance for primary outcomes (124/466; 26.6%) and secondary outcomes (108/466; 23.2%). Additionally, 21% of primary outcomes (98/466) were partly discordant. Half of the publications (233/466; 50.0%) reported only part of the registered 'secondary outcomes' without specifying reasons. Most registrations on ClinicalTrials.gov did not include 'Other Outcomes' (469/544; 86.2%), but of the 75 trials that did, only 12 (16%) showed complete concordance for these outcomes. Specific reasons for discordance in primary outcomes, such as COVID-19-related delays or enrolment issues, were cited in only four publications. A small minority of 12 publications provided explanations for the non-publication of some ‘secondary outcomes’ (e.g., published elsewhere). Nearly two million individuals (1,998,003) were either planned for enrolment or enrolled in the 544 registered interventional malaria trials on ClinicalTrials.gov. Of these, 1,526,081 people were enrolled in studies that were published. For the studies that were not published, plans had been made to enrol 417,922 participants. However, it is unclear if this enrolment occurred, as the results of these studies were not published.

**Table 3:** Concordance between published and registered outcomes

Outcome(s)	*(n)*	%
Primary outcome(s)		
Complete concordance	242	51.9
Complete discordance	124	26.6
Partial discordant	98	21.0
No primary outcome registered	2	0.4
Secondary outcome(s)		
Complete concordance	119	25.5
Complete discordance	108	23.2
Partial discordant	233	50.0
No secondary outcome registered	6	1.3
Other outcome(s)		
Complete concordance	12	2.6
Complete discordance	31	6.6
Partial discordant	33	7.1
No other outcome registered	390	83.7

Analysis of outcome reporting bias in peer-reviewed publications (*n* = 466, see Fig. 1, Flowchart)

## Discussion

Registered interventional malaria research significantly influences global health outcomes but also appears to be affected by widespread dissemination and outcome reporting biases, as this study highlights (Box 2). Alarmingly, 36% of registered interventional malaria studies from 2010 to 2020 were not published, indicating a substantial gap in the dissemination of medical knowledge. However, looking at it from another perspective, the publication rate for interventional malaria research (64%) is slightly higher than the average across various research fields. It has been reported that between 50 and 70% of studies registered on ClinicalTrials.gov are eventually published, while 30% to 50% remain unpublished [29], a finding corroborated by another analysis which indicated that, on average, 54% of studies registered in trial registries were published [30]. The reasons for the slightly higher publication rate of malaria trials are not clear, but it might be speculated that this field is less influenced by large commercial and pharmaceutical interests, often associated with 'blockbuster drugs,' as indicated by the rather low number of industry related funders/sponsors in the field of malaria research (see Table S1), which could potentially lead to reduced bias. This study also revealed that studies funded or sponsored by the industry were less likely to publish their peer-reviewed results within the WHO's 24-month timeframe. This tendency of industry-funded trials to be less frequently published is a recognized pattern, likely due to the suppression of unfavourable outcomes or results that are not commercially attractive [31]. A study that assessed the funding for clinical trials listed on clinicaltrials.gov revealed that the number of newly registered trials increased significantly, doubling from 9,321 in 2006 to 18,400 in 2014. During this period, the number of trials funded by industry rose by 1,965, marking a 43% increase. In contrast, trials funded by the National Institutes of Health saw a decline, decreasing by 328 trials, or 24% [32]. The reason why malaria clinical trials receive less funding than those for some other diseases is not entirely clear. However, it may be in great part due to limited commercial interest, as the primary affected populations are in low-income regions. Currently, malaria clinical trials receive funding from a diverse array of sources, including public sector entities, non-profit organizations, and private sector partnerships (table S1). Key funders include the National Institutes of Health (NIH), which supports various malaria research initiatives. Additionally, the European and Developing Countries Clinical Trials Partnership (EDCTP), backed by the European Union, plays a crucial role in financing these malaria trials.

Box 2. Key messages.

**Box 2:** Key messages

What is already known on this topic Non-timely or delayed dissemination and selective reporting of research outcomes can distort evidence, increasing the risk of bias, overestimating treatment effectiveness, and misrepresenting adverse effects A considerable proportion of medical research studies that are registered do not result in publication or get published with significant delay The World Health Organization has, since 2017, recommended the timely publication of research results: within 12 months in registries for non-peer-reviewed results, and within 24 months in peer-reviewed journals following the primary study's completion
What this study adds No peer-reviewed publications were found for 193 out of 544 interventional malaria trials registered on ClinicalTrials.gov from 2010 to 2020 Registered malaria research is not published in a timely manner, with the majority of trials not meeting the WHO's designated timeframe (12 months for posting summary results after the primary completion date and 24 months for a peer-reviewed publication after the primary completion data) Discrepancies between published and reported outcomes are frequent, signifying that reporting bias is very prevalent in the field of malaria research

For those malaria trials that disseminated results, the process often took a considerable amount of time after trial completion. Most malaria trials failed to meet WHO standards [28] for timely dissemination of results within 12 months (82%). More critically, only 29% of the trials published a peer-reviewed article within 24 months, which equates to 730 days. Given that the median time from study completion to publication in a peer-reviewed journal was 777 days, it is evident that most studies required more than the WHO-recommended 24 months to publish, indicating significant delays in reporting results. This finding contrasts with studies examining the timing of publications for randomized controlled trials in other fields registered on ClinicalTrials.gov, which found that the median duration from the primary completion date to the first public posting of results on ClinicalTrials.gov was 19 months, and publication in a peer-reviewed journal took 21 months (~ 640 days) [33]. It is important to note that the analysis covers the period from 2010 to 2020, while the WHO publication recommendations were issued in mid-2017. Studies registered before this date cannot be retrospectively assessed against guidelines that were established later. However, these findings reflect the current state of malaria research and provide valuable insight into the feasibility of adhering to the WHO publication recommendations. Although delays in publishing malaria research are pronounced, the differences compared to other fields might not be that substantial. Malaria research trials encounter distinct challenges primarily due to their settings, often situated in resource-limited regions such as numerous field sites across Africa. For example, diagnostics in malaria research can be both time- and labour-intensive. The interpretation of microscopy slides for malaria density requires multiple researchers, and resolving discrepancies through reconciliation is a lengthy process. Similarly, advanced diagnostic technologies such as PCR and whole-genome sequencing are often unavailable at research sites, necessitating the transfer of samples to reference laboratories, frequently located overseas, which adds further complexity and contributes to delays in the dissemination of results in malaria research. Only a quarter of malaria trials list the USA, Europe, and Australia as geographic references (see Table S1, supplementary file 1) with more than half of the trials conducted solely in Africa. These environments pose significant logistical and infrastructural constraints, which can hinder data collection and analysis processes. Additionally, the financial limitations of these trials, frequently funded by governmental bodies, academic institutions, or philanthropic organizations rather than industry stakeholders, may exacerbate these difficulties, potentially leading to delays.

This observation prompts an important question: Do the WHO’s standards for timely dissemination and publication sufficiently account for the practical challenges of reporting trials conducted under these conditions? Some may argue that WHO’s standards are too ambitious for reporting malaria trials conducted in resource-limited settings. However, the malaria research community also has responsibility for timely dissemination of results. Researchers and funding bodies could allocate more resources, including time and money, to the post-trial phase to ensure that findings are made publicly available as soon as possible. Enhanced investment in this area could help bridge the gap between the completion of trials and the dissemination of their outcomes, thereby improving the overall impact of malaria research.

Further compounding this issue, a substantial discrepancy was observed in the alignment between registered objectives and reported outcomes in published research, with nearly half of primary outcomes and over two-thirds of secondary outcomes exhibiting complete or partial discordance. In contrast, it has been determined that around 25% of randomized controlled trials in other research fields exhibit a discrepancy between the initially registered outcomes and the actual primary outcomes reported [3, 4, 7, 8, 34–37]. The reasons for this significant discrepancy in the studied malaria trials remain unclear. It seems unlikely that a more rigorous application of criteria, assessing whether published outcomes deviated from those pre-registered on ClinicalTrials.gov, can alone explain this divergence. Retrospective registration of clinical trials induces outcome reporting bias because it allows researchers to alter or select outcomes based on the data observed, rather than adhering to pre-specified objectives [38, 39].

The ideal practice is to perform trial registration before the start of the trial, yet about one-third of malaria trials were registered retrospectively with little change observed during the period 2015–2020 (Fig. S2, Supplementary file 1). It is known that the timing of registration, especially when done retrospectively, influences the choice of reported outcomes, thus introducing discrepancies and bias in the reported results [40]. Certainly, reported outcomes in publications may be modified to better align with specific intentions. This can be influenced by journals’ preferences for studies with more ‘exciting’ (positive) results, as well as incentives to publish favourable data on interventions, particularly drugs. This also considers the possible pressures to please funding bodies with positive results, thereby increasing the researchers’ reputation and chances of securing future funding [37]. However, these practices can raise concerns regarding the transparency and integrity of the reported results [38–40].

It is important to acknowledge that valid and justifiable reasons, such as unforeseen methodological challenges, evolving research priorities, or refined analytical approaches, might necessitate adjustments to originally registered outcomes. Notwithstanding this, such changes should be promptly updated and highlighted in the registration. The possibility of a justified, albeit unregistered, change in outcomes should be carefully evaluated before attributing all reporting biases to negative motives, as not all discrepancies suggest misconduct or poor research practices. Addressing the discordance between registered and published outcomes should be a collective effort by funders, researchers, editors, and peer-reviewers [18, 41]. A practical approach could involve submitting the trial registration alongside the final manuscript, ensuring that both are reviewed together. Editors could also be proactive in inquiring about reasons for any changes to better understand their validity. Another proposed option is to implement a two-stage review process, where editors and reviewers would be blinded to the results and the parts of the discussion that pertain to those results [42]. Funding agencies for malaria research could also contribute by actively implementing policies that encourage the timely dissemination of results [43].

Significant shortcomings in the registration and updating processes were noted, corroborating earlier studies that pointed to the inadequacy of registration quality on ClinicalTrials.gov [44, 45]. These issues contribute to potential outcome reporting biases, exacerbated by the flexibility in the registration process which allows for selective outcome reporting [46–49].

These observations also affected this study, as the accuracy and completeness of data on ClinicalTrials.gov were limiting factors. Trial statuses were sometimes outdated or incomplete, and because of exclusion criteria possibly some trials that were completed (but listed as ‘not yet recruiting’, ‘recruiting’, or ‘still active’) were excluded. Furthermore, the descriptions of primary and secondary outcomes were often vague, indicating a lack of stringent quality control during the registration process. However, it seems unlikely that this substantially influenced the overall outcome.

It should be noted that this study relied solely on ClinicalTrials.gov, excluding smaller (inter)-national registries, which may have led to the omission of some interventional malaria research. Furthermore, the applied methodology did not assess whether the nature of outcomes (positive or negative) influenced publication rates, nor were the reasons behind non-publication extensively explored beyond a limited email survey. Furthermore, delays in the trial and publication process caused by the SARS-CoV-2 pandemic affected the findings of this cross-sectional study.

To address publication and outcome reporting biases, the US/NIH and the European Commission have mandated clinical trial registration through platforms like ClinicalTrials.gov [8] and the EU Clinical Trials Register [50]. According to these regulations, results must be posted within one year after the primary completion date, with the FDA [4, 51] and European bodies enforcing penalties for delays, including fines [52, 53] and legal actions. However, implementation and compliance issues persist, especially in Europe where about 50% of trials fail to meet this deadline [53]. However, it may be unfeasible to expect the same standards from trials conducted in resource-limited settings such as Africa, where unique challenges and logistical constraints can hinder timely registration and reporting, exacerbating compliance difficulties. While mandatory publication and journals that publish negative results are seen as potential solutions to reduce bias [20], the disinclination to publish negative findings could affect journal impact factors [54]. That this issue is also relevant in malaria research, often conducted in poorer regions, is hinted at in the survey on unpublished trials, which indicated that negative or inconclusive results and safety issues are frequent yet underreported reasons for non-dissemination. However, the true dimension of these is unclear.

Delay or non-dissemination of malaria research hampers informed decision-making and compromises the integrity of the evidence base, patient care, and scientific trust and collaboration. A recent review on publication bias of COVID-19 trials recommends mandatory result reporting within ethics committee protocols, including clauses that stress timely publication and guidelines for committees to monitor publication timelines [55]. Along, a set of practical recommendations were proposed to overcome the problem of publication and dissemination bias in infectious diseases trials [55]. Ethically, it is imperative to honour the contributions of patients enrolled in clinical studies by ensuring (timely) publication of results. This study indicates that some 417,922 participants might have been involved in malaria trials which failed to disseminate their findings.

Improvements in registry practices and compliance with publication standards are necessary to mitigate these biases and address related ethical concerns [56]. Another option, for the long-term, is to ensure efficient and comprehensive access to clinical trial records by establishing a centralized, worldwide public portal to replace individual trial registries [57]. Research centres must incentivize timely publication and support researchers after the trial to ensure rapid result dissemination. Researchers are responsible for regularly updating trial registries, timely publishing, and using pre-print servers for swift dissemination. Additionally, malaria researchers could be encouraged to actively inform the WWARN Clinical Trials Publication Library about any malaria studies they have conducted, whether registered or unregistered, whether they have been published or not, including the reasons for non-dissemination or delayed dissemination.

## Conclusion

The prevalence of dissemination bias and outcome reporting bias in interventional malaria research is significant. Addressing these biases necessitates enhancing the quality of both registration and publication processes. Implementing measures to ensure timely research dissemination within WHO-recommended timeframes is crucial for enhancing transparency.

The findings from this study support the WHO's recommended timelines for disseminating results broadly. However, they also suggest that a more tailored approach may be necessary to address the specific challenges encountered in malaria research, particularly concerning trials conducted in resource-poor areas. All measures should benefit researchers, clinicians, and patients, and importantly uphold transparency, reproducibility, and ethical obligations toward participants in malaria research.

## Supplementary Information

Additional file 1. Extended methods and results section.

Additional file 2. Original downloaded data-set from ClinicalTrials.gov (date of download *15–11-2022*).

## Data Availability

All data generated or analysed during this study are included in this published article and its supplementary files. Supplementary file 2 contains the original downloaded excel dataset from www.clinicaltrials.gov (Date of download: 15 November 2022).
