# Supplementary material for: Dissemination and outcome reporting bias in clinical malaria intervention trials: a cross-sectional analysis
Source: Malar J. 2024 Sep 30;23:293. doi: 10.1186/s12936-024-05115-6 (PMC11443699; doi:10.1186/s12936-024-05115-6)
Supplement: Supplementary file 1 — Additional file 1. Extended methods and results section. [file 12936_2024_5115_MOESM1_ESM.docx]

**Supplementary file I**

***Supplementary (extended) methods*** *pag. 2*

1. *Identification of trials pag. 2*
2. *Study domain (Box 1) pag. 3*
3. *Identifying subsequent publications pag. 4*
4. *Variables – General pag. 8*
5. *Variables - Number of enrolled study participants pag. 10*
6. *Variable - Completion to publication time pag.11*
7. *Categorization of trial registries with dissemination and outcome reporting bias pag 12*
8. *Analysis of dissemination bias (A) pag. 13*
9. *Analysis of Outcome reporting bias (B) pag. 14*
10. *List of definitions in methods section pag. 16*

***Supplementary table S1*.** Malaria research registered at www.ClinicalTrials.gov and subsequent publication(s) pag. 20

***Supplementary figure S2****. Trends in Publication Status of Retrospectively Registered Malaria Trials pag. 23*

***Supplementary results:*** *Chi-square-tests funder/sponsor/geography pag. 24*

*Supplementary checklist STROBE for cross-sectional studies pag.26*

**1) Identification of trials:**

Registrations ‘first posted’ between 01 January 2010 - 01 January 2020 were selected, excluding registrations ‘not yet recruiting’, ‘recruiting’ ‘still active’, ‘enrolling by invitation’ trials and with completion date 01 July 2021 (estimated or actual). This range was chosen because, between 2000 and 2010, it was not mandatory to register interventional trials, though the recommendation/requirement was introduced around 2005 for most countries. We selected 2010 to allow sufficient time for the implementation of this requirement. The 2020 cut-off was chosen to provide ample time for the publication of trial results, allowing at least 30 months to publish registered studies. This ensures that if a study concluded in late 2023 and no publication is found, it wouldn't be misclassified as "non-disseminated" simply due to insufficient time for publication.. The included registrations contained ‘completed trials’, ‘withdrawn’, ‘terminated’, ‘suspended’ and ‘unknown’ status trials for all ages and sexes. These trials may offer valuable insights; for instance, a trial could be withdrawn due to safety concerns with a novel malaria drug, and excluding such information could lead to incomplete assessments and dissemination bias. The exact definitions of the afore mentioned labels can be found in on the website of ClinicalTrials.gov (https://prsinfo.ClinicalTrials.gov/definitions.html). The most used definitions are found below in the list of definitions. Five types of intervention types were included; ‘drug’: e.g. trials evaluating new drugs and/or versus placebo, ‘biological’: e.g. vaccine studies, ‘dietary’: e.g. vitamins and mineral supplementation trials, ‘behavioural/procedure/device’: for example lifestyle, counselling trials, procedures rapid diagnostic test and ‘other’: trials not fitting the previous defined groups.

The primary completion date (as registered in the trial registry) was used when the completion date was missing in the trial registry. The search results were examined to identify any doubts regarding the inclusion of a registration. In that case, the authors (LP and BJV) discussed the issue and resolved the issue by consensus.

**2) Study domain**

This study explores dissemination bias by analysing registered research which is subsequently published or not published (domains A and B, see box S1 which depicts the study field of dissemination bias).

**Supplementary box 1.** Study domain.

|  | *Publication (yes)* | *Publication (no)* |
| --- | --- | --- |
| *Registration (yes)* | *Registered and published (A)* | *Registered, not published (B)* |
| *Registration (no)* | *Not registered, but published (C)* | *Not registered, not published (D)* |

**3) Identifying subsequent publications**

The corresponding publications, matching the registrations, were identified in peer-reviewed biomedical journals by using ClinicalTrials.gov (ClinicalTrials.gov) reference. For an overview of the search process, see the flow diagram page 7. A search was conducted (by author LP) through PubMed, Google, Google Scholar, EMBASE, BING and Yahoo by using the Clinical Trial registration number (NCTXXXXX), title, name of the principal researcher, other contact person and/or the condition studied. Also, the WWARN Clinical Trials Publication Library was cross-checked to identify publications. In addition, ClinicalTrials.gov registrations were also checked to assess whether (non-peer reviewed) results were posted on the ClinicalTrials.gov website in the results section. Author BJV subsequently searched for registrations for which no peer-reviewed publication had been found by author LP. The authors finished this search at 6^th^ December 2022. After several updates, the latest update of the search was finished on 24 January 2024. In the last update of the search, also artificial intelligence assistants were used: ChatGPT version 4.0 (knowledge cut-off date April 2023) and Perplexity.ai (version beta release 0 (v0), January 2024). The prompt used for the search in Chat GPT and Perplexity.ai was as follows: “Please find the publication, full text, or paper associated with the NCT number [NCT number] and the title [Title of NCT registration], and provide URLs to the search results.”Additionally, a PubMed reverse search was conducted by author BJV. All PubMed-indexed published malaria clinical trials were identified, using the MesH term malaria. The search filter ‘clinical trial’ was used, and the custom range was set between 01-01-2018 and 01-01-2024. The PubMed search range was set to 2 years prior to our dataset (01-01-2010) due to the practice during that period of publishing results first and registering the trial later on ClinicalTrials.gov to meet formal requirements. All full text were assessed of the search results to assess whether they were related to a NCT registration. Sometimes it was rather difficult to establish whether a publication was actually related to the NCT registration; these issues were solved by consensus.

If no publication was identified, LP sent an email or a ResearchGate message to the first and/or corresponding author (contact information mentioned in the registration or checked with Google). In this correspondence, we also inquired about the reasons of non-publication or delays in publication. Some researchers were contacted regarding more than one registration number. If no response was received, we sent two additional e-mails at 7-10 day intervals, with a maximum of three emails. Emails regarding registrations that did not receive a response were categorized as unpublished.

Online pre-print servers such as medRxiv were not searched. We did not include MedRxiv in our search for publications as it was founded (June 2019) only six months prior to our cut-off date (1st of January 2020). If there was uncertainty about whether an identified study matched the registered trial, an e-mail was sent to one of the authors or contact for clarification.

**Flow diagram of subsequent publication of peer-reviewed publications:**

**
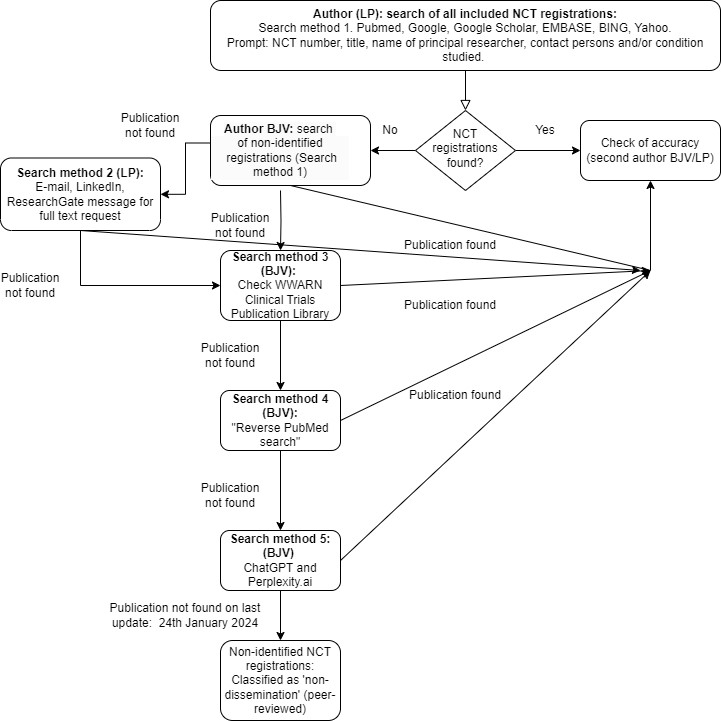
**

LP: Author Lydia Pool, BJV: Author Benjamin Jelle Visser.

**4) Variables - General**

The registrations were downloaded from clinicaltrials.gov as an excel file for analysis with study type interventional, all age groups, for all sexes, all status recruitments and all results (with or without study results). Further details are described in Supplement S1. Besides the NCT registration number, the registration database included the following characteristics ‘study status’, ‘type interventions’, ‘randomization’, ‘sponsor/collaborators’, ‘funder’, ‘outcomes’, ‘enrolment’, ‘age’, ‘location(s)’, ‘first posted’, ‘study start’, ‘primary completion’, ‘completion’, ‘results first posted’.

‘Timing of registration’ was established by comparing the registration date ‘first posted’ and the ‘start trial’ date and classified as 1.‘registered before’ (‘first posted’ before ‘start trial’) 2.’same month’ (‘first posted’/‘start trial’ same month/date) 3.‘in between’ (‘first posted’ after ‘start trial’ date and before ‘completion date’) 4.‘after completion’ (‘first posted’ in same month/after ‘completion date’).

In the ClinicalTrials.gov download, the field of ‘outcomes’ included all prespecified outcomes. The outcomes were separated into 3 different columns, the ‘primary’, ‘secondary’ and ‘other outcomes’ as formulated in the Study Record Detail (Beta.ClinicalTrial.gov registration files).

Type of ‘interventions’ was a pre-categorized variable labelled by ClinicalTrials.gov, consisting of ‘drug’, ‘biological’, ‘dietary’, procedure, behaviour, diagnostic, device and ‘other’. The types of studies ‘procedure’, ‘behaviour’, ‘diagnostic’ and ‘device’ were mainly focused on implementation and were grouped together. The majority of the ‘biological’ type of interventions consisted of malaria vaccine and adjuvants studies. The group of ‘others’ varied, e.g., a placebo study or a public health intervention for malaria. When a NCT registration number mentioned different types of interventions, the first intervention type was chosen.

The remaining variables ‘age population’, ‘enrolment’, ‘status trial’, ‘randomization’ and ‘funder’ were also pre-categorized by ClinicalTrials.gov and were followed as such. Enrolment was a continuous variable but converted into a categorical variable in this study. The sponsor was extracted from the combined sponsor/collaborator column and subdivided into the seven umbrella groups of sponsorship. The ‘locations’ of all the different trials were divided into nine different regions or combination of regions. If the trial location was missing in the download, any geographic information in the title was checked or locations in the registration were checked and adapted. The non-baseline characteristic ‘study duration’ (number of days) was calculated between registered ‘start study’ date and ‘completion date’ (the registered actual or estimated completion date). In instances where no completion date was specified, the registered ‘primary completion date’ was used.

**5. Variables - Number of enrolled study participants**

The total enrolment (n) of all published registrations was established by adding up all enrolments of the individual trials. The number of study participants of the non-published studies was not determined, because this number remains unknown. The intended number of enrolment of non-published studies could be calculated based on the registration data. These numbers of study participants were retrieved from studies meeting the inclusion criteria (and thus excluding ‘not yet recruiting, ‘recruiting’, ‘still active’ and ‘enrolling by invitation’ studies). We did not determine whether the CT.gov registered (planned) number of participants to be enrolled matched exactly with the actual enrolment number in the subsequent publication.

**6. Variable - Completion to publication time**

The median completion-to-publication time was calculated in a ‘raw’ version after establishing the (online first) publication date of the first publication per registration number. The ‘adjusted’ version after exclusion of the publications who were published before ‘registration’ or ‘completion’ and excluding the timing of registration after start trial (Timing category III and IV). Keeping in mind that registration after study completion frequently occurs to meet formal requirements (‘retrospective registration’).

The median start-publish time raw was calculated by adding up study duration time and completion-publish time. The ‘adjusted’ version also with excluding the negatives (published before ‘registration’ or ‘completion’) and excluding timing of registration after start trial (Timing category III and IV). The median study duration and IQR were calculated both for trials that resulted in publications and for trials that did not results in peer-reviewed publications.

**7. Categorization of trial registries with dissemination and outcome reporting bias**

To explore the bias, published (peer-reviewed) registrations were converted into a separate excel dataset, with all matching publications to each NCT registration number. If one NCT number resulted in one or multiple publications, they were all included. If a publication referenced multiple NCT numbers, it was not included in the analysis of outcome reporting bias. The reason for excluding such publications is that they drew data from multiple trials, making it non-feasible to determine any discrepancy between the reported outcomes and those of the corresponding registrations. For each published registration, the date of the initial publication, excluding study protocols, was gathered. This registered trials were analyzed to determine the number of entries which had dissemination bias (A) or which showed outcome reporting bias (B) (see figure 1: flow chart in main manuscript).

**8. Analysis of dissemination bias (A)**

The proportion (percentage) of non-dissemination (non-publication) and publication in registered interventional malaria clinical trials, the percentage of registrations meeting WHO dissemination standards. On May 18, 2017, the WHO issued a joint statement endorsing a recommendation that research results should be published within registries (non-peer-reviewed) within 12 months of the primary study's completion (defined as the last data collection time point for the last subject for the primary outcome measure) and within 24 months of study completion for publication of the research results in a peer-reviewed journal [1]. The ‘Timing of registration’ of the published registrations was defined as prospectively (category I or II) or retrospectively (category III and IV), see also Table 1. The linear regression model was employed to assess the impact of the WHO's 2017 endorsement on clinical trial registration rates and non-timely events before and after June 2017. The dependent variable (Y) was the registration rates and non-timely events, while the independent variable (X1) was the time period, coded as 0 for pre-June 2017 and 1 for post-June 2017. The analysis was performed using Python's *statsmodels* library (Python 3.11) (Ordinary Least Squares), with statistical significance determined at the 0.05 level. Missing data were handled by complete case analysis, excluding any cases with incomplete information. The regression model included an intercept to capture the mean values before June 2017, and coefficients were calculated to estimate the change after June 2017. The goodness-of-fit of the model was evaluated using R-squared and adjusted R-squared values, while diagnostic tests, including the Omnibus and Jarque-Bera tests, were performed to assess model assumptions of normality and autocorrelation (Durbin-Watson test)

**9. Outcome reporting bias (B)**

To assess outcome reporting bias, the primary-, secondary- and other outcomes in the registrations were compared with their corresponding publication(s) to identify any discordance between them. The identified publications which met our inclusion criteria were scored by comparing the pre-registered ‘primary’, ‘secondary’ and ‘other’ outcomes with the reported outcomes in the corresponding publications. The scoring possibilities were ‘complete discordance’, ‘complete concordance’, ‘partly concordance with reason’, ‘partly concordance without reason’ and ‘no pre-registered outcome’ In this study, we used strict definitions for concordance and discordance between pre-registered outcomes and published outcomes in malaria research.

- Complete concordance was defined as the pre-registered outcome aligning precisely with the published outcome, for example, when both registered and published outcomes were ‘mortality on day 28.

- Complete discordance was identified when there was no correspondence whatsoever between the pre-registered and published outcomes; for instance, the registered outcome being "mortality on day 28" while the published outcome was ’fever clearance time.’

- Partial concordance was considered when the pre-registered outcome only partially matched the published outcome, such as the registered outcome being ‘mortality on day 28’, and the published outcome indicating ‘mortality on day 48’. Furthermore, if multiple primary outcomes were pre-registered, and only a portion of them were reported, they were also categorized as ‘partial concordance’.

In this study, we refrained from evaluating the justifiability of reasons for changes in primary or secondary outcomes, as this is a subjective matter, and instead solely focused on discrepancies between publications and their corresponding registrations.

Discordance of the primary, secondary and other outcomes (n;%) were reported descriptively. Although we evaluated discrepancies between registered and published outcomes, our goal was not to individually list all publications that do not align with their corresponding trial registries. We refrained from this to avoid harming researchers' reputations and judging deviations from registered protocols, as there could be plausible and justifiable reasons for these differences that are unknown to us. This approach was chosen because it does not advance the objective of enhancing the quality of reporting and reducing outcome reporting bias.

1. **List of definitions**

The following list of definitions is fully following the glossary from www.Clinicaltrials.gov (https://clinicaltrials.gov/study-basics/glossary)

***Age or age group***

A type of eligibility criteria that indicates the age a person must be to participate in a clinical study. This may be indicated by a specific age or the following age groups:

The age groups are:

Child (birth-17)

Adult (18-64)

Older Adult (65+)

#### **Clinical trial**

Another name for an interventional study.

#### **ClinicalTrials.gov identifier (NCT number)**

The unique identification code given to each clinical study upon registration at ClinicalTrials.gov. The format is "NCT" followed by an 8-digit number (for example, NCT00000419).

#### **Collaborator**

An organization other than the sponsor that provides support for a clinical study. This support may include activities related to funding, design, implementation, data analysis, or reporting.

#### **Enrollment**

The number of participants in a clinical study. The "estimated" enrollment is the target number of participants that the researchers need for the study.

#### **First posted**

The date on which the study record was first available on ClinicalTrials.gov after National Library of Medicine (NLM) quality control (QC) review has concluded. There is typically a delay of a few days between the date the study sponsor or investigator submitted the study record and the first posted date.

#### **First submitted**

The date on which the study sponsor or investigator first submitted a study record to ClinicalTrials.gov. There is typically a delay of a few days between the first submitted date and the record's availability on ClinicalTrials.gov (the first posted date).

#### **Funder type**

Describes the organization that provides funding or support for a clinical study. This support may include activities related to funding, design, implementation, data analysis, or reporting. Organizations listed as sponsors and collaborators for a study are considered the funders of the study. ClinicalTrials.gov refers to four types of funders:

- U.S. National Institutes of Health
- Other U.S. Federal agencies (for example, Food and Drug Administration, Centers for Disease Control and Prevention, or U.S. Department of Veterans Affairs)
- Industry (for example: pharmaceutical and device companies)
- All others (including individuals, universities, and community-based organizations)

#### **Interventional study (clinical trial)**

A type of clinical study in which participants are assigned to groups that receive one or more intervention/treatment (or no intervention) so that researchers can evaluate the effects of the interventions on biomedical or health-related outcomes. The assignments are determined by the study's protocol. Participants may receive diagnostic, therapeutic, or other types of interventions.

#### **Last update posted**

The most recent date on which changes to a study record were made available on ClinicalTrials.gov. There may be a delay between when the changes were submitted to ClinicalTrials.gov by the study's sponsor or investigator (the last update submitted date) and the last update posted date.

#### **Last update submitted**

The most recent date on which the study sponsor or investigator submitted changes to a study record to ClinicalTrials.gov. There is typically a delay of a few days between the last update submitted date and when the date changes are posted on ClinicalTrials.gov (the last update posted date).

#### **Listed location countries**

Countries in which research facilities for a study are located. A country is listed only once, even if there is more than one facility in the country. The list includes all countries as of the last update submitted date; any country for which all facilities were removed from the study record are listed under removed location countries.

#### **Location terms**

In the search feature, the Location terms field is used to narrow a search by location-related terms other than Country, State, and City or distance. For example, you may enter a specific facility name (such as National Institutes of Health Clinical Center) or a part of a facility name (such as Veteran for studies listing Veterans Hospital or Veteran Affairs in the facility name). Note: Not all study records include this level of detail about locations.

#### **Outcome measure**

For clinical trials, a planned measurement described in the protocol that is used to determine the effect of an intervention/treatment on participants. For observational studies, a measurement or observation that is used to describe patterns of diseases or traits, or associations with exposures, risk factors, or treatment. Types of outcome measures include primary outcome measure and secondary outcome measure.

#### **Primary completion date**

The date on which the last participant in a clinical study was examined or received an intervention to collect final data for the primary outcome measure. Whether the clinical study ended according to the protocol or was terminated does not affect this date. For clinical studies with more than one primary outcome measure with different completion dates, this term refers to the date on which data collection is completed for all the primary outcome measures. The ‘estimated’ primary completion date is the date that the researchers think will be the primary completion date for the study.

#### **Primary outcome measure**

In a clinical study's protocol, the planned outcome measure that is the most important for evaluating the effect of an intervention/treatment. Most clinical studies have one primary outcome measure, but some have more than one.

#### **Recruitment status**

- **Not yet recruiting:** The study has not started recruiting participants.
- **Recruiting:** The study is currently recruiting participants.
- **Enrolling by invitation:** The study is selecting its participants from a population, or group of people, decided on by the researchers in advance. These studies are not open to everyone who meets the eligibility criteria but only to people in that particular population, who are specifically invited to participate.
- **Active, not recruiting:** The study is ongoing, and participants are receiving an intervention or being examined, but potential participants are not currently being recruited or enrolled.
- **Suspended:** The study has stopped early but may start again.
- **Terminated:** The study has stopped early and will not start again. Participants are no longer being examined or treated.
- **Completed:** The study has ended normally, and participants are no longer being examined or treated (that is, the last participant's last visit has occurred).
- **Withdrawn:** The study stopped early, before enrolling its first participant.
- **Unknown:** A study on ClinicalTrials.gov whose last known status was recruiting; not yet recruiting; or active, not recruiting but that has passed its completion date, and the status has not been last verified within the past two years.

#### **Results first posted**

The date on which summary results information was first available on ClinicalTrials.gov after National Library of Medicine (NLM) quality control (QC) review has concluded. There is typically a delay between the date the study sponsor or investigator first submits summary results information (the results first submitted date) and the results first posted date. Some results information may be available at an earlier date if Results First Posted with QC Comments.

#### **Secondary outcome measure**

In a clinical study's protocol, a planned outcome measure that is not as important as the primary outcome measure for evaluating the effect of an intervention but is still of interest. Most clinical studies have more than one secondary outcome measure.

#### **Sponsor**

The organization or person who initiates the study and who has authority and control over the study.

#### **Study completion date**

The date on which the last participant in a clinical study was examined or received an intervention/treatment to collect final data for the primary outcome measures, secondary outcome measures, and adverse events (that is, the last participant's last visit). The ‘estimated’ study completion date is the date that the researchers think will be the study completion date.

#### **Study start date**

The actual date on which the first participant was enrolled in a clinical study. The ‘estimated’ study start date is the date that the researchers think will be the study start date.

**Table S1. Malaria research registered at www.ClinicalTrials.gov and subsequent publication(s)**

|  | **Total number of malaria research registrations** | | **Timely dissemination** **(non peer-reviewed)**  *results in registry within WHO 12 months’ timeframe)* | | **Non-timely dissemination (non peer-reviewed)**  *no results in registry within WHO 12 months’ timeframe* | | **Timely publication (peer- reviewed journal)**  *Published within WHO 24 months’ timeframe* | | **Non-timely publication (peer-reviewed journal)**  Not published within WHO 24 months’ timeframe | | **Published (peer-reviewed) (total)**  *Published irrespective of timeframe* | | **Not published (total)**  *Not published before last search (Jan 2024)* | |
| --- | --- | --- | --- | --- | --- | --- | --- | --- | --- | --- | --- | --- | --- | --- |
| **Registrations** | | | | | | | | | | | | | | |
| Malaria research registered at clinicaltrials.gov (registrations) | 544 | 100.0% | 100 | 18.4% | 444 | 81.6% | 158 | 29.0% | 386 | 71.0% | 351 | 64.5% | 193 | 35.5% |
| **Clinical trial study status** | | | | | | | | | | | | | | |
| Completed | 457 | 84.0% | 85 | 85.0% | 372 | 83.8% | 142 | 89.9% | 315 | 81.6% | 316** | 90.0% | 141** | 73.1% |
| Terminated | 23 | 4.2% | 8 | 8.0% | 15 | 3.3% | 5 | 3.2% | 18 | 4.7% | 15 | 4.3% | 8 | 4.1% |
| Withdrawn | 19 | 3.5% | 1 | 1.0% | 18 | 4.0% | 1 | 0.6% | 18 | 4.7% | 1 | 0.3% | 18 | 9.3% |
| Suspended | 1 | 0.2% | 1 | 1.0% | 0 | 0.0% | 1 | 0.6% | 0 | 0.0% | 1 | 0.3% | 0 | 0.0% |
| Unknown | 44 | 8.1% | 5 | 5.0% | 39 | 8.9% | 9 | 5.7% | 35 | 9.0% | 18 | 5.1% | 26 | 13.5% |
| **Timing of registration** | | | | | | | | | | | | | | |
| Before start trial (cat. I) | 248 | 45.6% | 53 | 53.0% | 195 | 43.9% | 75 | 47.5% | 173 | 44.8% | 165 | 47.00% | 83 | 43.0% |
| Date same as start trial (cat. II) | 97 | 17.8% | 17 | 17.0% | 80 | 18.0% | 23 | 14.6% | 74 | 19.2% | 58 | 16.5% | 39 | 20.2% |
| After start trial ‘in between’)(cat. III) | 129 | 23.7% | 24 | 24.0% | 105 | 23.7% | 44 | 27.8% | 85 | 22.0% | 90 | 25.6% | 39 | 20.2% |
| After completion trial (cat. IV) | 70 | 12.9% | 6 | 6.0% | 64 | 14.4% | 16 | 10.1% | 54 | 14.0% | 38 | 10.8% | 32 | 16.6% |
| **Secondary outcomes** | | | | | | | | | | | | | | |
| Yes | 486 | 89.3% | 92 | 92.0% | 394 | 88.7% | 149* | 94.3% | 337* | 87.3% | 329** | 93.7% | 157** | 81.3% |
| No | 58 | 10.7% | 8 | 8.0% | 50 | 11.3% | 9 | 5.7% | 49 | 1.7% | 22 | 6.3% | 36 | 18.7% |
| **Other outcomes** | | | | | | | | | | | | | | |
| Yes | 75 | 13.8% | 10 | 10.0% | 65 | 14.6% | 22 | 15.7% | 53 | 13.7% | 55 | 15.7% | 173 | 89.6% |
| No | 469 | 86.2% | 90 | 90.0% | 379 | 85.4% | 136 | 84.3% | 333 | 86.3% | 296 | 84.3% | 20 | 10.4% |
| **Intervention type** | | | | | | | | | | | | | | |
| Drug | 323 | 59.4% | 66 | 66.0% | 257 | 57.9% | 97 | 61.4% | 226 | 58.5% | 215 | 61.2% | 108 | 56.0% |
| Biological | 118 | 21.7% | 20 | 20.0% | 98 | 22.1% | 30 | 19.0% | 88 | 22.8% | 68 | 19.4% | 50 | 25.9% |
| Dietary | 21 | 3.9% | 1 | 1.0% | 20 | 4.5% | 7 | 4.% | 14 | 3.6% | 16 | 4.6% | 5 | 2.6% |
| Other | 47 | 8.6% | 7 | 7.0% | 40 | 0.0% | 12 | 7.6% | 35 | 9.1% | 27 | 7.7% | 20 | 10.3% |
| Procedure/  Behaviour/Device | 35 | 6.4% | 6 | 6.0% | 29 | 6.5% | 12 | 7.6% | 23 | 6.0% | 25 | 7.1% | 10 | 5.2% |
| **Randomization** | | | | | | | | | | | | | | |
| Randomised | 391 | 71.9% | 76 | 76.0% | 315 | 64.2% | 121 | 76.6% | 270 | 69.9% | 267* | 76.1% | 124* | 64.2% |
| Not randomised | 153 | 28.1% | 24 | 24.0% | 129 | 35.8% | 37 | 23.4% | 116 | 30.1% | 84 | 23.9% | 69 | 35.8% |
| **Funder** | | | | | | | | | | | | | | |
| US government | 16 | 2.9% | 1 | 1.0% | 15 | 3.4% | 2* | 1.3% | 14* | 3.6% | 5 | 1.4% | 11 | 5.7% |
| NIH | 20 | 3.7% | 4 | 4.0% | 16 | 3.6% | 2 | 1.3% | 18 | 4.7% | 11 | 3.1% | 9 | 4.7% |
| Industry | 30 | 5.5% | 8 | 8.0% | 22 | 5.0% | 5 | 3.2% | 25 | 6.5% | 17 | 4.9% | 13 | 6.7% |
| other | 318 | 58.5% | 53 | 53.0% | 265 | 59.7% | 101 | 63.9% | 217 | 56.2% | 211 | 60.1% | 107 | 55.4% |
| Industry/Other | 86 | 15.8% | 18 | 18.0% | 68 | 15.3% | 19 | 12.0% | 67 | 17.4% | 54 | 15.4% | 32 | 16.6% |
| US/Other | 33 | 6.0% | 4 | 4.0% | 29 | 6.5% | 9 | 5.7% | 24 | 6.2% | 19 | 5.4% | 14 | 7.3% |
| NIH/Other | 25 | 4.6% | 8 | 8.0% | 17 | 3.8% | 14 | 8.8% | 11 | 2.8% | 21 | 6.0% | 4 | 2.1% |
| US/Ind | 8 | 1.5% | 1 | 1.0% | 7 | 1.6% | 1 | 0.6% | 7 | 1.8% | 7 | 2.0% | 1 | 0.5% |
| 3x Funder type | 8 | 1.5% | 3 | 3.0% | 5 | 1.1% | 5 | 3.2% | 3 | 0.8% | 6 | 1.7% | 2 | 1.0% |
| **Sponsor** | | | | | | | | | | | | | | |
| University | 237 | 43.6% | 43 | 43.0% | 194 | 43.7% | 88* | 55.7% | 149* | 38.6% | 169* | 48.2% | 68* | 35.2% |
| NGO | 24 | 4.4% | 3 | 3.0% | 21 | 4.7% | 6 | 3.8% | 18 | 4.7% | 17 | 4.8% | 7 | 3.6% |
| Army | 23 | 4.2% | 3 | 3.0% | 20 | 4.5% | 3 | 1.9% | 20 | 5.2% | 11 | 3.1% | 12 | 6.2% |
| Pharmacy | 85 | 15.6% | 21 | 21.0% | 64 | 14.4% | 19 | 12.0% | 66 | 17.1% | 57 | 16.2% | 28 | 14.5% |
| Government/National Institute | 83 | 15.3% | 17 | 17.0% | 66 | 14.9% | 23 | 14.5% | 60 | 15.5% | 53 | 15.1% | 30 | 15.6% |
| Individual researcher | 13 | 2.4% | 3 | 3.0% | 10 | 2.3% | 5 | 3.2% | 8 | 2.1% | 7 | 2.0% | 6 | 3.1% |
| Other | 79 | 14.5% | 10 | 10.0% | 69 | 15.5% | 14 | 8.9% | 65 | 16.8% | 37 | 10.6% | 42 | 21.8% |
| **Age** | | | | | | | | | | | | | | |
| Child | 124 | 22.8% | 24 | 24.0% | 100 | 22.5% | 44 | 27.8% | 80 | 20.7% | 84* | 23.9% | 40* | 20.7% |
| Adult | 204 | 37.5% | 29 | 29.0% | 175 | 39.4% | 44 | 27.8% | 160 | 41.4% | 119 | 33.9% | 85 | 44.0% |
| Older Adult | 1 | 0.2% | 0 | 0.0% | 1 | 0.2% | 0 | 0.0% | 1 | 0.3% | 0 | 0.0% | 1 | 0.5% |
| Child/Adult | 31 | 5.7% | 5 | 5.0% | 26 | 5.9% | 8 | 5.1% | 23 | 6.0% | 23 | 6.6% | 8 | 4.2% |
| Adult/Older Adult | 44 | 8.1% | 9 | 9.0% | 35 | 7.9% | 11 | 7.0% | 33 | 8.5% | 22 | 6.3% | 22 | 11.4% |
| Child/Adult/Older Adult | 140 | 25.7% | 33 | 33.0% | 107 | 24.1% | 51 | 32.3% | 89 | 23.1% | 103 | 29.3% | 37 | 19.2% |
| ***Anticipated enrolment*** | | | | | | | | | | | | | | |
| <100 | 244 | 44.9% | 33 | 33.0% | 211 | 47.5% | 51* | 32.3% | 193* | 50.0% | 131** | 37.3% | 113** | 58.6% |
| <500 | 153 | 28.1% | 33 | 33.0% | 120 | 27.0% | 56 | 35.4% | 97 | 25.1% | 109 | 31.0% | 44 | 22.8% |
| <1000 | 41 | 7.5% | 11 | 11.0% | 30 | 6.8% | 12 | 7.6% | 29 | 7.5% | 29 | 8.3% | 12 | 6.2% |
| <10.000 | 80 | 14.7% | 17 | 17.0% | 63 | 14.2% | 27 | 17.1% | 53 | 13.8% | 62 | 17.7% | 18 | 9.3% |
| >10.000 | 26 | 4.8% | 6 | 6.0% | 20 | 4.5% | 12 | 7.6% | 14 | 3.6% | 20 | 5.7% | 6 | 3.1% |
| ***Geography*** | | | | | | | | | | | | | | |
| West-Africa | 106 | 19.5% | 20 | 20.0% | 86 | 19.4% | 34 | 18.7% | 72 | 18.7% | 67* | 19.1% | 39* | 20.2% |
| Central Africa | 120 | 22.0% | 19 | 19.0% | 101 | 22.7% | 45 | 19.4% | 75 | 19.4% | 85 | 24.2% | 35 | 18.1% |
| South Africa | 32 | 5.9% | 10 | 10.0% | 22 | 5.0% | 8 | 6.2% | 24 | 6.2% | 23 | 6.5% | 9 | 4.7% |
| > 2 different African countries | 24 | 4.4% | 10 | 10.0% | 14 | 3.2% | 10 | 3.6% | 14 | 3.6% | 22 | 6.3% | 2 | 1.0% |
| Asia & Asia/Africa | 93 | 17.1% | 17 | 17.0% | 76 | 17.1% | 26 | 17.4% | 67 | 17.4% | 56 | 16.0% | 37 | 19.2% |
| South-America & S-A/Africa | 27 | 5.0% | 6 | 6.0% | 21 | 4.7% | 8 | 4.9% | 19 | 4.9% | 20 | 5.7% | 7 | 3.6% |
| US/Europe | 125 | 23.0% | 17 | 17.0% | 108 | 24.3% | 23 | 26.4% | 102 | 26.4% | 66 | 18.8% | 59 | 30.6% |
| Australia | 17 | 3.1% | 1 | 1.0% | 16 | 3.6% | 4 | 3.4% | 13 | 3.4% | 12 | 3.4% | 5 | 2.6% |
| **Study duration** | | | | | | | | | | | | | | |
| Missing completion date | 4 | 0.7% | 0** | 0.0% | 4** | 0.9% | 0** | 0.0% | 4** | 1.0% | 2** | 0.6% | 2** | 1.0% |
| < 1 year | 175 | 32.2% | 12 | 12.0% | 163 | 36.7% | 30 | 19.0% | 145 | 37.6% | 90 | 25.6% | 85 | 44.1% |
| < 2 year | 188 | 34.6% | 30 | 30.0% | 158 | 35.6% | 52 | 32.9% | 136 | 35.2% | 128 | 36.5% | 60 | 31.1% |
| < 3 year | 90 | 16.5% | 19 | 19.0% | 71 | 16.0% | 33 | 20.9% | 57 | 14.8% | 66 | 18.8% | 24 | 12.4% |
| > 3 year | 87 | 16.0% | 39 | 39.0% | 48 | 10.8% | 43 | 27.2% | 44 | 11.4% | 65 | 18.5% | 22 | 11.4% |

**Figure S2. Trends in Publication Status of Retrospectively Registered Malaria Trials**

*Legend* This figure displays the publication trends for malaria trials registered on ClinicalTrials.gov from 2010 to 2020 (n=544). Of these, 345 trials (63%) were registered prospectively. The remaining 199 trials (37%) were registered retrospectively. The figure categorizes these retrospectively registered trials into those with disseminated results (grey bars) and those without (open bars), shown annually.

**Supplementary results: Chi-square-tests:**

Timely publication versus non-timely publication (WHO-24 month timeframe) for peer-reviewed journal publications.

**Sponsor: Industry versus non-industry for timely publication of peer-reviewed publications.**

|  | Timely publication (WHO-24 months) | Non-timely publication (WHO 24-months) | ***Row Totals*** |
| --- | --- | --- | --- |
| Industry (sponsor) | 25  (36.01)  [3.37] | 99  (87.99)  [1.38] | 124 |
| Non-industry (sponsor) | 133  (121.99)  [0.99] | 287  (298.01)  [0.41] | 420 |
| ***Column Totals*** | 158 | 386 | **544**  **(Total)** |

The chi-square statistic is 6.1493. The p-value is .013146. The result is significant at p < .05.

**Funder: Industry versus non-industry for timely publication of peer-reviewed publications.**

|  | Timely publication (WHO-24 months) | Non-timely publication (WHO 24-months) | ***Row Totals*** |
| --- | --- | --- | --- |
| Industry (funder) | 19  (24.69)  [1.31] | 66  (60.31)  [0.54] | 85 |
| Non-industry (funder) | 139  (133.31)  [0.24] | 320  (325.69)  [0.10] | 459 |
| ***Column Totals*** | 158 | 386 | **544**  **(Total)** |

The chi-square statistic is 2.1886. The p-value is .139037. The result is not significant at p < .05.

**Geography: Africa versus rest of the world for timely publication of peer-reviewed publications.**

|  | Timely publication (WHO-24 months) | Non-timely publication (WHO 24-months) | ***Row Totals*** |
| --- | --- | --- | --- |
| Africa | 97  (81.90)  [2.78] | 185  (200.10)  [1.14] | 282 |
| Rest of the world | 61  (76.10)  [2.99] | 201  (185.90)  [1.23] | 262 |
| ***Column Totals*** | 158 | 386 | **544**  **(Total** |

The chi-square statistic is 8.1415. The p-value is .004326. The result is significant at p < .05.

|  | Timely publication (WHO-24 months) | Non-timely publication (WHO 24-months) | ***Row Totals*** |
| --- | --- | --- | --- |
| Drug/biological | 127  (128.08)  [0.01] | 314  (312.92)  [0.00] | 441 |
| Non-Drug/biological | 31  (29.92)  [0.04] | 72  (73.08)  [0.02] | 103 |
| ***Column Totals*** | 158 | 386 | **544**  **(Total)** |

The chi-square statistic is 0.0684. The p-value is .793744. The result is not significant at p < .05.

|  | Dissemination | Non-dissemination | ***Row Totals*** |
| --- | --- | --- | --- |
| Cat I and II | 223  (222.60)  [0.00] | 122  (122.40)  [0.00] | 345 |
| Cat II and IV | 128  (128.40)  [0.00] | 71  (70.60)  [0.00] | 199 |
| ***Column Totals*** | 351 | 193 | **544**  **(Grand Total)** |

The chi-square statistic is 0.0055. The p-value is .940839. The result is not significant at p < .05.

|  | **Timely publication (WHO-24 months)** | **Non-timely publication (WHO 24-months)** | ***Row Totals*** |
| --- | --- | --- | --- |
| **Small and medium sized trials** | **107  (115.31)  [0.60]** | **290  (281.69)  [0.24]** | **397** |
| **Large trials** | **51  (42.69)  [1.62]** | **96  (104.31)  [0.66]** | **147** |
| ***Column Totals*** | **158** | **386** | **544  (Grand Total)** |

The chi-square statistic is 3.1199. The p-value is .077342. The result is not significant at p < .05.

STROBE Statement—Checklist of items that should be included in reports of ***cross-sectional studies***

|  | Item No | Recommendation | Page in manuscript (M) / supplementary file (S) |
| --- | --- | --- | --- |
| **Title and abstract** | 1 | (*a*) Indicate the study’s design with a commonly used term in the title or the abstract | M page 2 |
|  |  | (*b*) Provide in the abstract an informative and balanced summary of what was done and what was found | M page 2 |
| Introduction | | |  |
| Background/rationale | 2 | Explain the scientific background and rationale for the investigation being reported | M page 5-6 |
| Objectives | 3 | State specific objectives, including any prespecified hypotheses | M page 6 |
| Methods | | |  |
| Study design | 4 | Present key elements of study design early in the paper | M page 6-10 |
| Setting | 5 | Describe the setting, locations, and relevant dates, including periods of recruitment, exposure, follow-up, and data collection | S page 2-4 |
| Participants | 6 | (*a*) Give the eligibility criteria, and the sources and methods of selection of participants | S page 2 |
| Variables | 7 | Clearly define all outcomes, exposures, predictors, potential confounders, and effect modifiers. Give diagnostic criteria, if applicable | S page 8-11 |
| Data sources/ measurement | 8* | For each variable of interest, give sources of data and details of methods of assessment (measurement). Describe comparability of assessment methods if there is more than one group | S page 5-7 |
| Bias | 9 | Describe any efforts to address potential sources of bias | S page 5-15 |
| Study size | 10 | Explain how the study size was arrived at | M page 35 |
| Quantitative variables | 11 | Explain how quantitative variables were handled in the analyses. If applicable, describe which groupings were chosen and why | S page 8-11 |
| Statistical methods | 12 | (*a*) Describe all statistical methods, including those used to control for confounding | S page 13-15 |
|  |  | (*b*) Describe any methods used to examine subgroups and interactions | S page 13-15 |
|  |  | (*c*) Explain how missing data were addressed | S page 13-15 |
|  |  | (*d*) If applicable, describe analytical methods taking account of sampling strategy | n/a |
|  |  | (*e*) Describe any sensitivity analyses | S page 13-15 |
| Results | | |  |
| Participants | 13* | (a) Report numbers of individuals at each stage of study—eg numbers potentially eligible, examined for eligibility, confirmed eligible, included in the study, completing follow-up, and analysed | M page 10, 35 |
|  |  | (b) Give reasons for non-participation at each stage | n/a |
|  |  | (c) Consider use of a flow diagram | M page 35 |
| Descriptive data | 14* | (a) Give characteristics of study participants (eg demographic, clinical, social) and information on exposures and potential confounders | M page 10-13 |
|  |  | (b) Indicate number of participants with missing data for each variable of interest | Table S1, S page 20-23 |
| Outcome data | 15* | Report numbers of outcome events or summary measures | M page 11 |
| Main results | 16 | (*a*) Give unadjusted estimates and, if applicable, confounder-adjusted estimates and their precision (eg, 95% confidence interval). Make clear which confounders were adjusted for and why they were included | M Page 10-13, S page 20-23 |
|  |  | (*b*) Report category boundaries when continuous variables were categorized | M Page 10-13, S page 20-23 |
|  |  | (*c*) If relevant, consider translating estimates of relative risk into absolute risk for a meaningful time period | n/a |
| Other analyses | 17 | Report other analyses done—eg analyses of subgroups and interactions, and sensitivity analyses | S page 24-25 |
| Discussion | | |  |
| Key results | 18 | Summarise key results with reference to study objectives | M page 13-15 |
| Limitations | 19 | Discuss limitations of the study, taking into account sources of potential bias or imprecision. Discuss both direction and magnitude of any potential bias | M page 15-18 |
| Interpretation | 20 | Give a cautious overall interpretation of results considering objectives, limitations, multiplicity of analyses, results from similar studies, and other relevant evidence | M page 13-20 |
| Generalisability | 21 | Discuss the generalisability (external validity) of the study results | M page 19 |
| Other information | | |  |
| Funding | 22 | Give the source of funding and the role of the funders for the present study and, if applicable, for the original study on which the present article is based | n/a |

*Give information separately for exposed and unexposed groups.

**Note:** An Explanation and Elaboration article discusses each checklist item and gives methodological background and published examples of transparent reporting. The STROBE checklist is best used in conjunction with this article (freely available on the Web sites of PLoS Medicine at http://www.plosmedicine.org/, Annals of Internal Medicine at http://www.annals.org/, and Epidemiology at http://www.epidem.com/). Information on the STROBE Initiative is available at www.strobe-statement.org.

**References**

1. Joint statement on public disclosure of results from clinical trials [Internet]. [cited 2024 Apr 19]. Available from: https://www.who.int/news/item/18-05-2017-joint-statement-on-registration
